# The Green Parkinson’s Belt: agricultural regions associated with increased Parkinson’s disease burden in Mexico

**DOI:** 10.3389/fnagi.2026.1832314

**Published:** 2026-05-28

**Authors:** Hector R. Martinez, Francisco A. Luna-Rangel, Brenda Gonzalez-Bedolla, Cecilio Armengol-Garcia, Daniel Rebolledo-Garcia, Beatriz L. Rodriguez, Jose A. Figueroa-Sanchez

**Affiliations:** 1 Tecnologico de Monterrey, School of Medicine and Health Sciences, Monterrey, Nuevo León, Mexico; 2 Instituto de Neurologia y Neurocirugia, Centro Medico Zambrano Hellion, TecSalud, San Pedro Garza Garcia, Nuevo Leon, Mexico; 3 Clínica de Disautonomías y Movimientos Anormales, Santa Fe, Mexico; 4 John A Burns School of Medicine, University of Hawaii at Manoa, Honolulu, HI, United States

**Keywords:** agriculture, environmental exposure, epidemiology, Parkinson’s disease, pesticides

## Abstract

**Background:**

Parkinson’s disease (PD) is projected to increase substantially in Mexico over coming decades, yet its geographic distribution has not been systematically examined at the state level. This study aimed to evaluate whether PD prevalence in Mexico exhibits geographic clustering, with particular attention to regions characterized by intensive agricultural and industrial activity.

**Methods:**

We conducted an ecological cross-sectional study using publicly available state-level data from Mexico covering the period 2015–2024. PD case counts were obtained from the national epidemiological surveillance system, and population denominators were derived from the 2020 national census. State-level prevalence was calculated per 100,000 inhabitants as annual averages across the study period. States were grouped into a Northern Hotspot, a Western Hotspot, and remaining states based on geographic clustering patterns and agricultural productivity profiles. Geographic inequality was assessed using the Gini index, Theil index, and coefficient of variation. Regional differences were evaluated with proportion tests and Poisson regression models with population offset, reported as prevalence ratios (PRs) with 95% confidence intervals (CIs).

**Results:**

PD prevalence showed substantial geographic heterogeneity across Mexican states over the 2015–2024 period. Colima recorded the highest average prevalence (38.8 per 100,000), followed by Durango (21.4), Sinaloa (14.9), Morelos (14.8), and Chihuahua (13.7 per 100,000). Inequality metrics confirmed marked dispersion in state-level prevalence (Gini 0.419; Theil 0.312; coefficient of variation 0.949). Average prevalence was higher in the Western Hotspot (8.6 per 100,000; PR 1.54, 95% CI 1.44–1.63) and the Northern Hotspot (13.8 per 100,000; PR 2.57, 95% CI 2.42–2.74) compared with the remaining states (4.9 per 100,000).

**Conclusion:**

PD prevalence in Mexico exhibits pronounced geographic heterogeneity, with distinct clustering observed in northern and western states. These patterns are consistent with a non-random spatial distribution and highlight regions where agricultural, industrial, and environmental exposures may warrant further investigation. Given the ecological design and important limitations including the absence of age standardization and variability in healthcare access across states, these findings are hypothesis-generating and do not permit causal inference. They underscore the need for more robust epidemiological surveillance, standardized case ascertainment, and individual-level studies to better characterize the determinants of PD distribution in Mexico.

## Introduction

In 2021, 11.77 million people worldwide were living with Parkinson’s disease (PD), according to the Global Burden of Disease Project (Luo et al., 2025). By 2050, this figure is projected to reach 25.2 million a rise driven primarily by population aging, with an estimated global prevalence of 267 cases per 100,000 inhabitants (Su et al., 2025). The increase has been disproportionately pronounced in low- and middle-income countries, where demographic aging, industrialization, and expanding diagnostic capacity have amplified disease burden (Ben-Shlomo et al., 2024). Mexico is projected to become one of only two countries, alongside Indonesia, to enter the global top ten for PD burden by 2050, with an estimated increase of 171% a trajectory that underscores the country’s accelerated epidemiological transition (Su et al., 2025).

The association between environmental exposures and PD has been recognized since 1983, when the structural similarity between MPTP and paraquat was first described (Langston et al., 1983). Since then, multiple studies have documented increased PD risk associated with exposure to paraquat, maneb, rotenone, and organophosphates, particularly in settings involving agricultural water and soil contamination (Wang et al., 2011; Barbeau et al., 1987; Breckenridge et al., 2016; Tangamornsuksan et al., 2019). Paraquat induces dopaminergic injury through oxidative stress and mitochondrial dysfunction, replicating key mechanisms implicated in neurodegeneration (See et al., 2022; Ossowska et al., 2006). Beyond pesticides, trichloroethylene and air pollution have also been associated with elevated PD risk. Notably, many of these compounds including paraquat, maneb, carbamates, benomyl, and glyphosate retain active regulatory authorizations in Mexico (Cofepris, 2026), underscoring the continued relevance of environmental exposures in regions with intensive agricultural activity.

Agriculture represents one of Mexico’s principal productive sectors. Tequila production is restricted to *Agave tequilana* cultivated within the Denomination of Origin zone centered in Jalisco, while export avocados must originate from certified orchards in Michoacán and, more recently, Jalisco (Rojas and Schaefer, 2024). These regulatory frameworks concentrate high-intensity cultivation and the associated use of pesticides implicated in PD within specific geographic regions, making them particularly relevant for epidemiological investigation.

Prior studies have identified geographic heterogeneity in PD burden within Mexico. Rodríguez-Violante et al. analyzed national incidence data from 2014 to 2017 and reported higher rates in Jalisco and Colima, with intermediate values in Michoacán (Scielo, 2022). Building on these observations, Martínez-Ramírez et al. (2020) introduced the concept of a “Parkinson’s Belt” in Mexico, identifying a northern hotspot and elevated prevalence in Colima and Jalisco. However, neither study specifically examined whether this geographic clustering corresponded to regions of intensive agricultural production or region-specific productive activities such as tequila agave, avocado, and lime cultivation.

Despite growing recognition of environmental determinants of PD, no ecological analysis has formally evaluated whether the spatial distribution of PD in Mexico corresponds to regions of intensive agricultural activity. The objective of this study is therefore to examine whether the western region of Mexico exhibits a higher burden of PD in relation to its agricultural productivity, while also characterizing geographic clustering patterns across other regions of the country. We hypothesize that high-density production zones particularly those associated with tequila agave and avocado cultivation may be linked to state-level geographic clustering of PD.

## Methods

### Study design and setting

This investigation was designed as a national, ecological, cross-sectional study using publicly available, aggregated state-level data from Mexico’s 32 federal entities. The study was designed, conducted, and reported in accordance with the Strengthening the Reporting of Observational Studies in Epidemiology (STROBE) guidelines for observational research (STROBE, n.d.). The unit of analysis was the federal state, and the study period encompassed the years 2015 through 2024. Statistical analyses were conducted in December 2025 following data consolidation and verification.

### Parkinson’s disease case data

State-level Parkinson’s disease case counts were obtained from publicly available records maintained by the General Directorate of Epidemiology through the National Epidemiological Surveillance System (SINAVE), operated by the Secretariat of Health of Mexico and integrated within the Unified Epidemiological Surveillance Information System (SUIVE) (Gob Mx, 2024). Cases were identified using the International Classification of Diseases, Tenth Revision (ICD-10) code G20. The surveillance system compiles case notifications reported by healthcare facilities across the national territory, including public hospitals, clinics, and primary care centers affiliated with the Mexican Social Security Institute (IMSS) and the Institute for Social Security and Services for State Workers (ISSSTE). Case registration follows a standardized cascade reporting process: a diagnosing physician registers the case at the healthcare unit using standardized SUIVE forms (SUIVE-1, SUIVE-2, and SUIVE-3); reports are subsequently forwarded from the local health jurisdiction to state health authorities for validation before being incorporated into the national epidemiological database. Aggregated, anonymized data are then released through the SUIVE platform as open-access surveillance statistics (Gob Mx, 2024).

The analysis covered the period 2015–2024. To reduce year-to-year variability inherent to passive surveillance systems and to obtain a stable estimate of the underlying disease burden, an annual average number of reported cases was calculated for each state across the full study period, and prevalence estimates were subsequently derived from these temporally smoothed figures. Because data are released in aggregated and anonymized form, no individual-level patient identifiers are present, and institutional ethical review was therefore not required. A recognized limitation of surveillance-based data is the inability to verify whether standardized diagnostic criteria for Parkinson’s disease were consistently applied across reporting facilities or providers, which may introduce uncertainty regarding diagnostic validity.

### Population denominators: 2020 population and housing census

Population denominators were derived from the 2020 Population and Housing Census (Censo de Población y Vivienda 2020), conducted by the National Institute of Statistics and Geography (INEGI) the most recent national census available at the time of this analysis (INEGI, 2020). The census constituted a national, cross-sectional, exhaustive enumeration exercise following a de jure approach, wherein all individuals were enumerated at their habitual place of residence as of the census reference date of 00:00 h on March 15, 2020. The target population encompassed all habitual residents within the national territory, including occupants of private and collective dwellings.

Data collection employed a multimodal strategy: the primary modality was direct face-to-face interview conducted via mobile computing devices, supplemented by internet-based self-enumeration (Computer Assisted Website Interview, CAWI) and telephone-assisted interviews (Computer Assisted Telephone Interview, CATI). Two structured instruments were deployed: a Basic Questionnaire of 38 items for exhaustive enumeration and an Extended Questionnaire of 103 items applied to a probabilistic sample of approximately four million dwellings to capture greater thematic depth. The census captured population-level variables including age, sex, health service affiliation, functional disability (assessed using the Washington Group methodology), and socioeconomic characteristics. Statistical results are disseminated at the national, state (Área Geoestadística Estatal AGEE), municipal (Área Geoestadística Municipal AGEM), and Basic Geostatistical Area (AGEB) levels, integrated within the National Geostatistical Framework (Marco Geoestadístico Nacional). For the present analysis, state-level population totals were used as denominators for prevalence calculations. A notable methodological limitation was the emergence of the COVID-19 pandemic, which necessitated the suspension of field activities on March 31, 2020 and the cancellation of the planned Post-Enumeration Survey intended to directly measure coverage; a compensatory verification phase was subsequently conducted between June and August 2020 to complete enumeration of pending dwellings without altering the original reference date. State-level demographic and socioeconomic characteristics including total population, proportion of females, proportion of individuals aged over 65 years, mean years of education, and percentage with healthcare coverage are presented in Table 1. Notably, the statistical model was not adjusted for age. This decision reflects a fundamental methodological constraint: the population denominators were derived from the 2020 INEGI census, whereas case counts originated from SINAVE surveillance records; these two data sources do not correspond to the same individuals. Applying individual-level age adjustment under these circumstances would be methodologically inappropriate, as it would assume a degree of data linkage that does not exist between the census and surveillance systems.

**Table 1:** State-level demographic characteristics in Mexico (2020), including total population, sex distribution, proportion aged ≥65 years, mean years of schooling, and healthcare coverage, derived from the National Population and Housing Census (INEGI).

State	Total population, n	Female population, %	Population aged 65 years or older, %	Mean years of schooling	Population with health care coverage, %
Aguascalientes	1,425,607	51.1	6.8	9.98	81.4
Baja California	3,769,020	49.6	6.5	9.56	77.1
Baja California Sur	798,447	49.2	6.1	10.00	83.2
Campeche	928,363	50.8	7.5	9.00	77.5
Chiapas	5,543,828	51.2	6.3	7.40	66.7
Chihuahua	3,741,869	50.5	7.5	9.48	84.4
Ciudad de México	9,209,944	52.2	11.1	11.02	72.6
Coahuila de Zaragoza	3,146,771	50.3	7.3	9.99	80.7
Colima	731,391	50.7	8.4	9.52	82.8
Durango	1,832,650	50.6	7.8	9.26	74.6
Guanajuato	6,166,934	51.4	7.6	8.55	79.0
Guerrero	3,540,685	52.0	8.9	7.77	74.3
Hidalgo	3,082,841	51.9	8.6	8.79	69.7
Jalisco	8,348,151	50.9	8.2	9.43	69.9
México	16,992,418	51.4	7.4	9.61	66.3
Michoacán de Ocampo	4,748,846	51.4	8.9	7.94	62.2
Morelos	1,971,520	51.8	9.6	9.59	71.9
Nayarit	1,235,456	50.4	8.9	9.40	77.7
Nuevo León	5,784,442	50.0	7.6	10.12	80.9
Oaxaca	4,132,148	52.2	9.6	7.70	70.3
Puebla	6,583,278	52.0	7.8	8.60	70.6
Querétaro	2,368,467	51.2	6.7	9.97	79.1
Quintana Roo	1,857,985	49.6	4.4	9.91	73.5
San Luis Potosí	2,822,255	51.4	9.0	9.14	82.5
Sinaloa	3,026,943	50.6	8.9	9.74	80.9
Sonora	2,944,840	50.0	8.0	9.94	81.2
Tabasco	2,402,598	51.1	7.2	9.18	68.5
Tamaulipas	3,527,735	50.8	8.1	9.55	79.5
Tlaxcala	1,342,977	51.6	7.4	9.33	71.8
Veracruz de Ignacio de la Llave	8,062,579	52.0	10.0	8.35	72.3
Yucatán	2,320,898	50.9	8.7	8.90	78.0
Zacatecas	1,622,138	51.2	8.7	8.52	79.7

### Agricultural exposure data: 2022 agricultural census

State-level agricultural activity data were obtained from the 2022 Agricultural Census (Censo Agropecuario 2022), the ninth national census of its kind, conducted by INEGI as a cross-sectional, exhaustive enumeration of Mexico’s agricultural, livestock, and forestry sectors (INEGI, 2022). The target population consisted of agricultural production units (Unidades de Producción Agropecuaria UPA), poultry production units, and forestry production units distributed across the national territory. The census reference period for agricultural and forestry production was the 2022 agricultural year (October 2021 to September 2022), with the livestock inventory reference date established as September 15, 2022.

Data collection was conducted from September 19 to November 30, 2022, via direct interview using mobile computing devices equipped with integrated digital cartography and specialized data-entry systems. Three structured instruments were deployed: a Basic Questionnaire for small and medium producers, an Extended Questionnaire for large producers, and a specialized Forestry Questionnaire. The census captured data across multiple domains, including total and cultivated land surface area (in hectares), crop types and production volumes, irrigation sources and water quality, and the use of agricultural inputs notably chemical fertilizers and pesticides (herbicides, insecticides, and fungicides). Data quality was maintained through online validation during capture, normalization of regional measurement units, application of 136 logical consistency criteria for the basic questionnaire and 207 for the extended version, and satellite imagery verification of declared land surface areas. The census achieved a final coverage rate of 98.66% of the national censusable territory. Results are disseminated at national, state, municipal, and locality levels within the National Geostatistical Framework, enabling spatial analysis at multiple administrative scales.

### Outcome variable

The primary outcome was the state-level crude prevalence of Parkinson’s disease, expressed as the annual average number of reported cases per 100,000 inhabitants, with the 2020 census population serving as the denominator. The use of temporally smoothed, period-averaged case counts was intended to mitigate the instability of year-specific estimates attributable to fluctuations inherent in passive surveillance reporting and to provide a more representative measure of the underlying disease burden across the study period.

### Exposure classification and hotspot definition

The primary exposure variable was regional classification, operationalized through the *a priori* identification of potential Parkinson’s disease hotspots at the state level. Hotspot classification was informed by state-level total agricultural surface area (hectares), obtained from the 2022 Agricultural Census (Table 2). This variable was selected as an ecological proxy for cumulative agricultural exposure intensity, on the premise that the overall scale of cultivated land reflects the plausible geographic distribution of agricultural inputs including pesticides with known or suspected dopaminergic neurotoxicity at the population level. It must be emphasized, however, that agricultural surface area does not directly quantify specific exposures, such as pesticide type, application rates, or individual-level contact, and must therefore be interpreted strictly as an ecological indicator subject to the limitations of aggregate-level inference; no causal attribution between agricultural activity and Parkinson’s disease risk is implied by this classification.

**Table 2:** State-level agricultural surface area in Mexico.

Agricultural surface area by state in Mexico (2020)	
State	Agricultural surface (ha)
Veracruz	2,572,357
Jalisco	1,472,007
Chiapas	1,443,989
Tamaulipas	1,436,961
Zacatecas	1,316,609
Sinaloa	1,281,178
Chihuahua	1,153,942
Michoacan	1,089,191
Puebla	1,051,870
Oaxaca	1,035,223
Guerrero	948.152
Guanajuato	928.099
Durango	809.741
San Luis Potosi	743.128
Sonora	652.924
State of Mexico	619.62
Yucatan	614.7
Hidalgo	517.064
Nuevo Leon	500.541
Nayarit	461.576
Tabasco	427.042
Campeche	401.314
Coahuila	238.179
Quintana Roo	235.057
Tlaxcala	230.484
Baja California	188.921
Morelos	170.266
Queretaro	161.81
Colima	133.735
Aguascalientes	130.704
Baja California Sur	37.409
Mexico City	18.134

The table presents total agricultural surface area (hectares) by state, derived from the National Agricultural Census, providing a proxy measure of agricultural activity relevant to the study’s exposure definition.

Based on this *a priori* spatial framework, states were assigned to one of three mutually exclusive categories: Northern Hotspot, Western Hotspot, and Other States. The Northern Hotspot region corresponds to a geographic cluster of elevated Parkinson’s disease prevalence in northern Mexico previously characterized by Martínez-Ramírez et al. (2020). The “Green Parkinson’s Belt,” encompassing the Western Hotspot, represents an original analytical contribution of the present study, derived from the observation of spatially contiguous elevated Parkinson’s disease prevalence across states characterized by intensive, export-oriented agricultural production particularly *agave tequilana* and avocado cultivation in western Mexico.

### Data sources and healthcare system context

In Mexico, epidemiological surveillance operates within a structurally fragmented healthcare system that shapes how cases are identified, diagnosed, and ultimately reported. The diagnostic pathway typically begins at the primary care level whether through community health posts, general practitioners, or outpatient clinics where initial clinical evaluation occurs. Patients requiring further investigation are referred to secondary-care hospitals, and those with conditions demanding specialist input or advanced diagnostics are escalated to tertiary-level centers, where definitive diagnosis is most reliably established. For neurological conditions in particular, confirmatory diagnosis frequently depends on specialist evaluation by neurologists, whose clinical presence is concentrated almost exclusively at this tertiary tier. National epidemiological data are aggregated through surveillance platforms, principally SINAVE (Sistema Nacional de Vigilancia Epidemiológica) and SUIVE (Sistema Único de Información para la Vigilancia Epidemiológica), which compile case notifications submitted across participating institutions. However, the fidelity of this surveillance is contingent on institutional reach and reporting capacity, both of which vary substantially across the system (Dantés et al., 2011).

The public sector is itself divided into parallel subsystems primarily IMSS, ISSSTE, and the SSa-affiliated state network each maintaining separate administrative and clinical infrastructure, with limited interoperability. The private sector, which serves a distinct segment of the population, operates largely outside national surveillance frameworks, representing an additional source of case under-ascertainment. Access to specialized diagnostic resources is markedly unequal: tertiary care facilities are geographically concentrated in the three largest metropolitan areas Mexico City, Monterrey, and Guadalajara leaving populations in rural and southern states with substantially reduced access to specialist services. This geographic asymmetry, compounded by socioeconomic barriers and institutional fragmentation, introduces heterogeneity in diagnostic ascertainment that is not uniformly distributed across the country. As a result, national surveillance data likely underestimate disease burden in marginalized and geographically peripheral populations, a structural limitation with direct implications for the interpretation of incidence and prevalence estimates derived from these sources (Dantés et al., 2011).

### Statistical analysis

All 32 states with complete annual case counts and population denominators in official sources for the study period were included; no individual eligibility criteria were applied, consistent with the ecological study design. To characterize the geographic inequality in Parkinson’s disease distribution, three complementary dispersion measures were calculated: the Gini index, the Theil index, and the coefficient of variation (CV) (Harper et al., 2013; Laporte, 2002). The Gini index was selected for its interpretability and established use in quantifying overall distributional inequality; the Theil index, grounded in information theory, permits decomposition of total inequality into within- and between-group components, thereby enabling a more granular assessment of spatial heterogeneity; and the CV provides a scale-independent measure of relative dispersion that complements these formal inequality statistics. Because reliance on a single metric may obscure distinct aspects of distributional heterogeneity, the concurrent use of all three was intended to improve analytical robustness. Between-group differences in prevalence were assessed using two-sided proportion tests comparing each hotspot region against the remaining states. To estimate the magnitude of regional excess prevalence while accounting for variation in state population size, Poisson regression models with a log link function and an offset term for the natural logarithm of the state population were fitted using robust variance estimation; results are expressed as prevalence ratios (PRs) with 95% confidence intervals (CIs). A two-sided *p < 0.05* was considered statistically significant. All analyses were conducted using R version 4.4.2.

## Results

All prevalence estimates and regional comparisons presented below are based on annual averages across the 2015–2024 study period.

**Figure 1:**
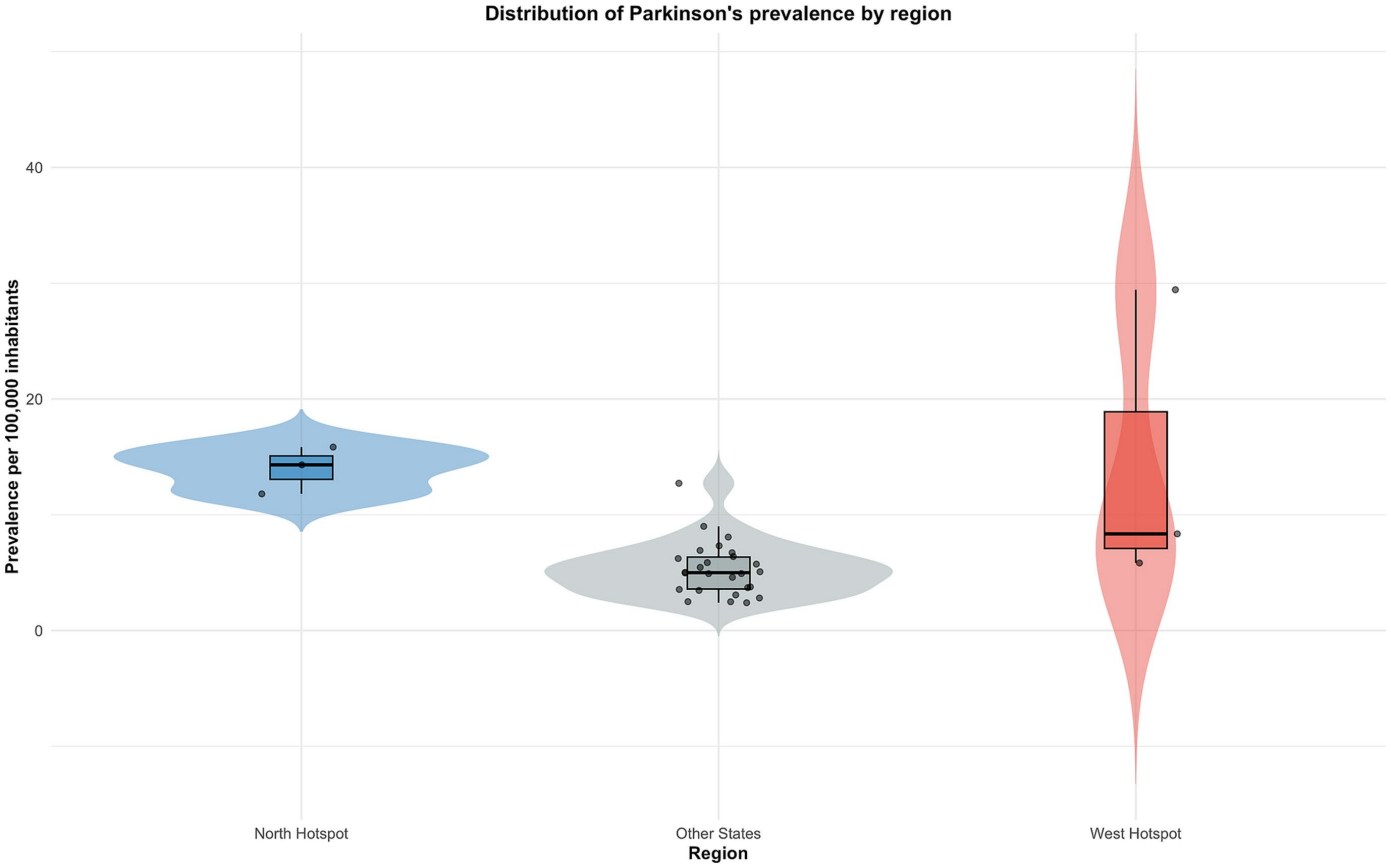
Geographic distribution of Parkinson’s disease hotspots in Mexico (2015–2024). Map illustrating regional clustering of Parkinson’s disease prevalence across Mexico. Blue circles represent states in the Northern Hotspot, and red circles represent states in the Western Hotspot (Green Parkinson’s Belt). Circle size is proportional to the state-level crude prevalence of Parkinson’s disease per 100,000 inhabitants.

State-level Parkinson’s disease prevalence exhibited marked geographic variation. Colima recorded the highest prevalence at 38.8 cases per 100,000 inhabitants (284 cases; population 731,391), followed by Durango (21.4 per 100,000), Sinaloa (14.9), Morelos (14.8), and Chihuahua (13.7 per 100,000). Tamaulipas, Campeche, Veracruz, Michoacán, and Jalisco showed prevalences ranging from 8.7 to 12.8 per 100,000. Inequality metrics across this period reflected substantial distributional heterogeneity, with a Gini index of 0.419, a Theil index of 0.312, and a coefficient of variation of 0.949 for prevalence rates; absolute case counts followed a comparable pattern (Gini 0.418, Theil 0.285, coefficient of variation 0.798). These patterns are illustrated in Figure 1.

**Figure 2:**
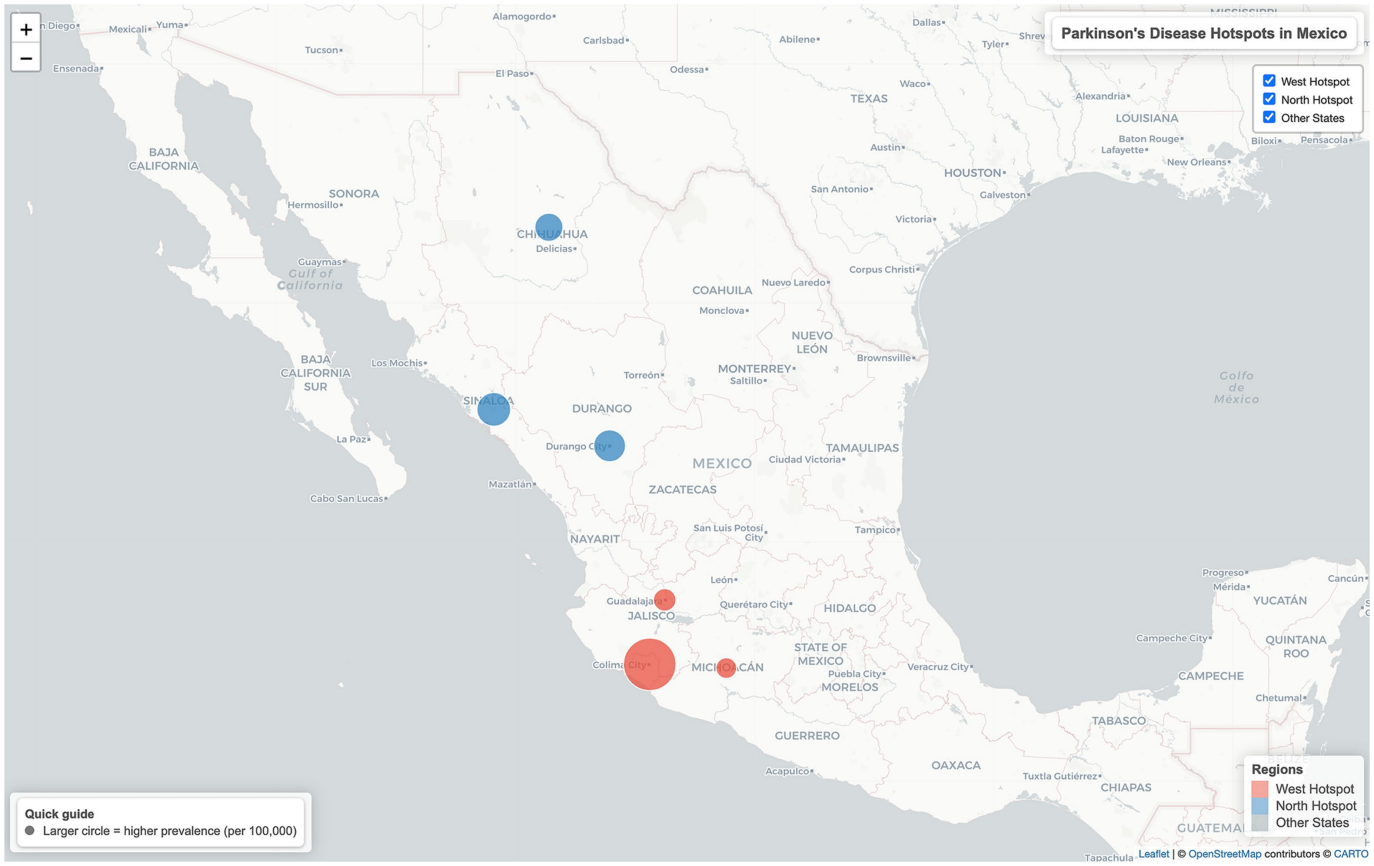
Distribution of Parkinson’s disease prevalence by region in Mexico (2015–2024). Violin and box plots showing the distribution of state-level crude prevalence of Parkinson’s disease (cases per 100,000 inhabitants) across three regions: Northern Hotspot, Western Hotspot, and Other States. Points represent individual states, while box plots indicate the median and interquartile range. The Western Hotspot shows the greatest variability, whereas the Northern Hotspot demonstrates consistently higher prevalence compared with other regions. Map data compiled from OpenStreetMap under the Open Data Commons Open Database License (ODbl), https://www.openstreetmap.org/copyright.

In the regional analysis, the Northern Hotspot recorded an average of 1,184 cases annually in a population of 8,601,462 inhabitants (≈13.8 per 100,000), and the Western Hotspot reported 1,189 cases in 13,828,388 inhabitants (≈8.6 per 100,000), compared with 5,094 cases in 103,584,174 inhabitants (≈4.9 per 100,000) in the remaining states. These geographic patterns are depicted in Figure 2.

Proportion tests indicated differences between each hotspot region and the remaining states. The Western Hotspot prevalence of 8.6 per 100,000 exceeded that of the remaining states (4.9 per 100,000) by 3.7 per 100,000 (*p < 0.001*). The Northern Hotspot exceeded the remaining states by an absolute difference of 8.0 to 9.6 per 100,000 (95% CI; *p < 0.001*) and was approximately 2.8 times higher than in the remaining states.

In Poisson regression models adjusted for state population, residence in the Western Hotspot was associated with a prevalence ratio (PR) of 1.54 (95% CI: 1.44–1.63; *p < 0.001*) relative to the remaining states, and residence in the Northern Hotspot with a PR of 2.57 (95% CI: 2.42–2.74; *p < 0.001*). Prevalence in the Western and Northern Hotspots was thus 54 and 157% higher, respectively, than in the rest of the country, consistent with geographic clustering of Parkinson’s disease prevalence across Mexico.

## Discussion

This study examined the geographic distribution of Parkinson’s disease prevalence across Mexican states using national surveillance data from 2015 to 2024. The analysis identified pronounced heterogeneity in state-level prevalence rates, with inequality metrics including the Gini index, Theil index, and coefficient of variation confirming that this dispersion exceeds what would be expected from random variation alone. Regional analyses identified two geographic clusters of elevated prevalence: a Northern Hotspot and a Western Hotspot, both showing prevalence rates substantially higher than the remaining states. Poisson regression models, adjusted for state population, yielded prevalence ratios of 1.54 (95% CI: 1.44–1.63) and 2.57 (95% CI: 2.42–2.74) for the Western and Northern Hotspots, respectively, relative to the rest of the country. These findings are consistent with a non-random spatial concentration of Parkinson’s disease within Mexico and corroborate previously reported geographic patterns, including the “northern epidemiological belt” described by Martínez-Ramírez et al. (2020) using data from 2014 to 2018.

The observed geographic clustering is consistent with the hypothesis that certain states share environmental, occupational, or industrial characteristics that may be associated with elevated local Parkinson’s disease risk. However, these patterns must be interpreted with considerable caution. The ecological design precludes any inference about individual-level exposure or risk, and the associations identified here are strictly descriptive. The convergence of multiple plausible environmental exposures including agricultural pesticide use, industrial solvent activity, and atmospheric pollution across high-prevalence regions suggests that the geographic distribution of Parkinson’s disease in Mexico may reflect an overlap of contributors rather than a single dominant factor. These interpretations remain speculative and are not demonstrated by the current analysis.

Several regional environmental characteristics provide plausible, hypothesis-generating context for the observed patterns, though none can be considered demonstrated by this study. In the Northern Hotspot, Chihuahua and Ciudad Juárez form one of the largest automotive manufacturing corridors in the country, encompassing 440 industrial plants distributed across more than 3,500 hectares (Bribiescas Silva et al., 2012). This manufacturing activity has historically involved chlorinated solvents in metal degreasing processes, making occupational exposure to trichloroethylene plausible a compound whose association with Parkinson’s disease is well established, though direct environmental measurements are not available for this region (Liu et al., 2025; Krzyzanowski et al., 2025; Dorsey et al., 2023; Martinez et al., 2026). In Durango, large-scale bean and corn production ranking the state as the third largest bean producer in Mexico involves pesticides including cypermethrin, imidacloprid, malathion, and fomesafen, which may contribute to sustained environmental and occupational exposure (Arnaiz, 2025; Jáquez-Matas et al., 2022). The regional economy is further supported by mining and metal-mechanic industries, which may augment exposure to industrial pollutants. Lázaro-Figueroa et al. similarly reported elevated Parkinson’s disease incidence in this region and suggested a potential contribution of pesticide and contaminated water exposure associated with local agricultural economies (Lázaro-Figueroa et al., 2023). Sinaloa, also part of the Northern Hotspot, is the leading tomato producer in Mexico, with more than 1,039,000 metric tons produced in 2017 and over 8,000 of the approximately 25,000 nationally cultivated hectares of protected tomato agriculture located within the state (Flores and Edwards, 2019; Amarillas, 2017). Tomato production has been proposed as a proxy for intensive pesticide use and a hypothesized risk factor for Parkinson’s disease (Sage, 1989; Bogers et al., 2026), and this agricultural profile may partly account for the elevated prevalence observed in the state.

The potential role of air pollution warrants consideration in specific states. Morelos, which exhibited elevated prevalence in this analysis, borders Mexico City one of the most polluted urban areas in the country. Neuropathological studies in young Mexico City residents have documented early changes compatible with Parkinson’s disease, including deposits of *α*-synuclein, hyperphosphorylated tau, β-amyloid, and TDP-43 in up to 23% of cases (Calderón-Garcidueñas et al., 2017; Calderón-Garcidueñas et al., 2022; Riojas-Rodríguez et al., 2016). Environmental monitoring in Morelos conducted in 2007 and 2009 confirmed the presence of PM10 and PM2.5 at multiple urban and industrial sites (Murata et al., 2022; Salcedo et al., 2012). Prior epidemiological studies have linked PM2.5 exposure to an increased Parkinson’s disease risk, with a reported odds ratio of 1.23 (95% CI 1.11–1.35); however, these associations should be interpreted cautiously given incomplete adjustment for confounders including age, smoking, and pesticide exposure, as well as heterogeneity in pollution sources and particulate composition across studies (Krzyzanowski et al., 2024; Salimi et al., 2020; Shin et al., 2018).

Our study extends prior geographic evidence by identifying a Western Hotspot encompassing Jalisco, Michoacán, and Colima a region we designate the Green Belt of Parkinson’s Disease, reflecting the convergence of intensive agricultural activity and potential pesticide exposure. This designation represents a hypothesis requiring prospective validation and should not be interpreted as a causal attribution. According to the 2022 Agricultural Census (INEGI, 2022), agave, avocado, and lime cultivation have undergone substantial expansion over the past 15 years agave from 155,080 to 361,475 hectares, avocados from 121,044 to 290,820 hectares, and limes from 148,782 to 367,931 hectares with this intensification concentrated in these three states. These crops are associated with the routine application of pesticides previously implicated in Parkinson’s disease risk. In avocado production, paraquat, glyphosate, and benomyl are used to manage fungal and arthropod pests (Fischer et al., 2019; Esparza-Jiménez et al., 2024). In *Agave tequilana* cultivation, parathion, paraquat, and glyphosate are applied against a range of soil and foliar pests (Fregoso-Zamorano et al., 2023). In citrus crops, glyphosate and paraquat serve as broad-spectrum herbicides (Alcántara-de La Cruz et al., 2019; Panozzo et al., 2020). Environmental monitoring of the Ayuquila-Armería river basin in Jalisco and Colima detected pesticides in 66% of surface water samples, including glyphosate, paraquat, atrazine, carbofuran, and diazinon (Rodríguez Aguilar et al., 2019), and a descriptive clinical study in Michoacán found that 36.1% of patients with Parkinson’s disease reported occupational pesticide exposure (García-Villa et al., 2022). Findings from other settings are consistent with this hypothesis: Priyadarshi et al. (2001) reported a pooled OR of 2.16 for Parkinson’s disease associated with pesticide exposure in United States studies, and Paul et al. (2024) found that residential or occupational proximity to paraquat applications in California’s Central Valley was associated with more than twice the likelihood of developing Parkinson’s disease (occupational OR 2.15, 95% CI 1.46–3.19; intensity-based OR 2.08, 95% CI 1.31–3.38; residential OR 1.91, 95% CI 1.30–2.83). These external findings are suggestive but cannot be extrapolated directly to the Mexican context without individual-level data.

Colima warrants specific comment. Its population of 731,391 inhabitants (INEGI, 2020) is substantially smaller than neighboring states, which may amplify rate instability through small-number bias. Nevertheless, population size alone is unlikely to fully account for its elevated prevalence, given the state’s intensive lime cultivation and documented pesticide use factors that may represent an additional environmental contribution independent of demographic artifact.

Several methodological limitations constrain the interpretation of these findings and must be considered collectively. The ecological design introduces the risk of ecological fallacy: associations observed at the aggregate level cannot be attributed to individuals, and unmeasured confounding at the state level cannot be excluded. The crude prevalence estimates were not adjusted for age or sex two major determinants of Parkinson’s disease risk because demographic stratification was not available from surveillance sources. As shown in Table 1, the proportion of individuals aged 65 years or older varies considerably across states, ranging from 4.4% in Quintana Roo to 11.1% in Mexico City, a difference that could meaningfully influence crude estimates and partially account for observed geographic disparities independent of any environmental exposure. Healthcare coverage also varies markedly across states (Table 1), ranging from approximately 62% in Michoacán to over 84% in Chihuahua; states with greater coverage and more developed specialist infrastructure are likely to achieve higher diagnostic ascertainment for a condition requiring neurological confirmation, introducing systematic ascertainment bias that may inflate prevalence in better-resourced regions. Surveillance systems such as SINAVE and SUIVE may additionally be subject to heterogeneous reporting quality across institutions and geographic areas. Agricultural surface area functions only as a proxy for pesticide exposure, and no direct environmental measurements of agrochemicals were available. Taken together, these limitations suggest that a portion of the observed geographic variation may reflect structural differences in population aging, healthcare access, and reporting capacity rather than true differences in disease burden, and they collectively preclude causal inference.

In conclusion, this study identifies pronounced geographic heterogeneity in Parkinson’s disease prevalence across Mexico, with distinct clustering in northern and western states that persists after accounting for population size. The observed patterns are consistent with a non-random spatial distribution and generate hypotheses regarding the potential contribution of agricultural, industrial, and atmospheric exposures in high-prevalence regions. These hypotheses are not demonstrated by the current analysis and must be evaluated in studies capable of individual-level exposure assessment. Strengthening environmental surveillance, standardizing Parkinson’s disease reporting across institutions, and conducting studies incorporating biomarkers of pesticide exposure, longitudinal environmental measurements, and high-resolution spatial models will be essential to advance causal understanding of the geographic patterns identified here.

## Conclusion

This ecological analysis demonstrates that Parkinson’s disease prevalence in Mexico is not uniformly distributed across states, but instead clusters geographically into two regions, a Northern Hotspot and a Western Hotspot, characterized by distinctive agricultural, industrial, and sociodemographic profiles. While the ecological design precludes causal inference, and important methodological constraints including the absence of age standardization, differential healthcare access, and heterogeneous surveillance quality limit the interpretation of these patterns, the findings represent the most comprehensive population-level characterization of Parkinson’s disease distribution in Mexico currently achievable with available national data. The observed spatial heterogeneity generates hypotheses warranting investigation in studies capable of individual-level exposure assessment, but does not, in itself, establish the determinants of regional variation.

These findings also underscore a broader and more immediate need: the strengthening of Mexico’s epidemiological data infrastructure. The limitations encountered in this analysis including the absence of age- and sex-stratified surveillance data, inconsistent reporting quality across states, gaps in healthcare coverage that affect diagnostic ascertainment, and the lack of integration between clinical, demographic, and environmental registries reflect structural deficiencies that constrain not only research on Parkinson’s disease but population-level health monitoring more broadly. Developing more robust, standardized, and interoperable epidemiological databases; improving case capture across institutions and regions; and reducing disparities in access to specialist diagnosis will be essential prerequisites for generating the evidence needed to understand and address the geographic distribution of chronic neurological disease in Mexico. Until such infrastructure is in place, population-level patterns derived from existing surveillance systems should be interpreted as provisional and hypothesis-generating rather than definitive estimates of disease burden.

## Data Availability

The raw data supporting the conclusions of this article will be made available by the authors, without undue reservation.
