# Supplementary material for: The Green Parkinson’s Belt: agricultural regions associated with increased Parkinson’s disease burden in Mexico
Source: Front Aging Neurosci. 2026 May 28;18:1832314. doi: 10.3389/fnagi.2026.1832314 (PMC13253685; doi:10.3389/fnagi.2026.1832314)
Supplement: Supplementary file 1 [file Data_Sheet_1.DOCX]

**STROBE Checklist**

**The Green Parkinson’s Belt: Agricultural Regions Associated with Increased Parkinson’s Disease Burden in Mexico**

| **Item** | **Recommendation** | **Description in manuscript** | **Location in manuscript** |
| --- | --- | --- | --- |
| **TITLE AND ABSTRACT** | | | |
| 1 | Title and abstract | (a) Indicate the study design with a commonly used term in the title or abstract. (b) Provide an informative and balanced summary of what was done and what was found. | Title: "The Green Parkinson’s Belt: Agricultural Regions Associated with Increased Parkinson’s Disease Burden in Mexico"; ecological study design stated in Abstract (Methods section). |
| **INTRODUCTION** | | | |
| 2 | Background / Rationale | Explain the scientific background and rationale for the investigation being reported. | Introduction, paragraphs 1–4: global PD burden, environmental risk factors, Mexico’s agricultural context, prior regional evidence (refs 1–14). |
| 3 | Objectives | State specific objectives, including any prespecified hypotheses. | Introduction, final paragraph: objective to examine geographic clustering of PD in relation to agricultural productivity; hypothesis stated regarding high-density production zones. |
| **METHODS** | | | |
| 4 | Study design | Present key elements of study design early in the paper. | Methods – Study Design and Setting: “national, ecological, cross-sectional study” using state-level data from Mexico’s 32 federal entities. |
| 5 | Setting | Describe the setting, locations, and relevant dates, including periods of recruitment, exposure, follow-up, and data collection. | Methods – Study Design and Setting: national study covering all 32 Mexican states, 2015–2024, statistical analyses conducted December 2025. |
| 6 | Participants | Cross-sectional study: Give the eligibility criteria, and the sources and methods of selection of participants. | Methods – Statistical Analysis: all 32 states with complete annual case counts and population denominators were included; no individual eligibility criteria applied (ecological design). |
| 7 | Variables | Clearly define all outcomes, exposures, predictors, potential confounders, and effect modifiers. Give diagnostic criteria, if applicable. | Methods – Outcome Variable: state-level crude PD prevalence per 100,000 (annual average, 2015–2024). Methods – Exposure Classification: regional classification (Northern Hotspot, Western Hotspot, Other States) based on agricultural surface area. Methods – Population Denominators: state demographic and socioeconomic covariates (Table 1). |
| 8 | Data sources / measurement | For each variable of interest, give sources of data and details of methods of assessment (measurement). Describe comparability of assessment methods if there is more than one group. | Methods: PD cases from SINAVE/SUIVE (ICD-10 G20) [ref 16]; population denominators from 2020 INEGI Census [ref 17]; agricultural exposure from 2022 Agricultural Census [ref 18]; healthcare system context [ref 19]. |
| 9 | Bias | Describe any efforts to address potential sources of bias. | Methods – Parkinson’s Disease Case Data: temporal smoothing (annual averages) to reduce year-to-year surveillance variability. Methods – Population Denominators: COVID-19 impact on census field activities acknowledged. Discussion – Limitations paragraph: ecological fallacy, ascertainment bias, differential healthcare access, age structure variability, surveillance heterogeneity discussed. |
| 10 | Study size | Explain how the study size was arrived at. | Methods – Statistical Analysis: all 32 states (complete national coverage) included; no sample size calculation applicable given census-level ecological design. |
| 11 | Quantitative variables | Explain how quantitative variables were handled in the analyses. If applicable, describe which groupings were chosen and why. | Methods – Outcome Variable: continuous prevalence expressed as annual average cases per 100,000. Methods – Exposure Classification: three categorical regional groups defined a priori. Methods – Statistical Analysis: Gini, Theil, CV as dispersion metrics [refs 20, 21]. |
| 12 | Statistical methods | (a) Describe all statistical methods, including those used to control for confounding. (b) Describe any methods used to examine subgroups and interactions. (c) Explain how missing data were addressed. (d) Cross-sectional study: if applicable, describe analytical methods taking account of sampling strategy. (e) Describe any sensitivity analyses. | (a) Proportion tests (two-sided) and Poisson regression with population offset (robust variance) for regional comparisons; Gini, Theil, CV for inequality. (b) Three pre-specified regional groups (Northern Hotspot, Western Hotspot, Other States). (c) No missing data; all 32 states had complete records. (d) Ecological design; unit of analysis is the state. (e) Sensitivity analyses not performed; acknowledged as limitation. |
| **RESULTS** | | | |
| 13 | Participants | (a) Report numbers of individuals at each stage of study. (b) Give reasons for non-participation at each stage. (c) Consider use of a flow diagram. | Results, first paragraph: all 32 Mexican states included; no exclusions. Methods – Statistical Analysis: inclusion criteria stated. |
| 14 | Descriptive data | (a) Give characteristics of study participants and information on exposures and potential confounders. (b) Indicate number of participants with missing data for each variable of interest. | Table 1: state-level total population, % female, % aged ≥65 years, mean years of education, healthcare coverage (2020 INEGI Census). Table 2: agricultural surface area by state. No missing data. |
| 15 | Outcome data | Cross-sectional study: Report numbers of outcome events or summary measures. | Results, paragraphs 1–3: state-level prevalence rates reported (top states listed); regional case counts and prevalence for Northern Hotspot (1,184 cases; 13.8/100,000), Western Hotspot (1,189 cases; 8.6/100,000), and remaining states (5,094 cases; 4.9/100,000). Inequality metrics (Gini, Theil, CV) reported. |
| 16 | Main results | (a) Give unadjusted estimates and, if applicable, confounder-adjusted estimates. (b) Report category boundaries when continuous variables were categorized. (c) If relevant, consider translating estimates of relative risk into absolute risk. | (a) Crude prevalence ratios from Poisson regression: Western Hotspot PR 1.54 (95% CI 1.44–1.63; p<0.001); Northern Hotspot PR 2.57 (95% CI 2.42–2.74; p<0.001) vs remaining states. (b) Categorical exposure: three regional groups. (c) Absolute differences reported: Western Hotspot 3.7/100,000 excess; Northern Hotspot 8.0–9.6/100,000 excess. |
| 17 | Other analyses | Report other analyses done e.g. subgroup analyses, sensitivity analyses. | No sensitivity analyses performed; acknowledged as a limitation in Discussion. |
| **DISCUSSION** | | | |
| 18 | Key results | Summarise key results with reference to study objectives. | Discussion, first paragraph: summary of geographic heterogeneity findings, inequality metrics, and PRs for both hotspot regions referenced to study objective. |
| 19 | Limitations | Discuss limitations of the study, taking into account sources of potential bias or imprecision. Discuss both direction and magnitude of any potential bias. | Discussion – Limitations paragraph: ecological fallacy, absence of age/sex standardization, differential healthcare access (Table 1), heterogeneous surveillance quality (SINAVE/SUIVE), agricultural surface area as proxy only, temporal mismatch between epidemiological data (2015–2024) and census denominators (2020). |
| 20 | Interpretation | Give a cautious overall interpretation of results considering objectives, limitations, multiplicity of analyses, results from similar studies, and other relevant evidence. | Discussion, paragraphs 2–6: cautious interpretation framed as hypothesis-generating; regional hypotheses (agriculture, solvents, air pollution) presented as speculative; comparison with prior studies (Martínez-Ramírez et al. [ref 14], Rodríguez-Violante et al. [ref 13]). |
| 21 | Generalisability | Discuss the generalisability (external validity) of the study results. | Discussion – Limitations paragraph and Conclusions: findings described as provisional and hypothesis-generating; causal inference precluded; context-specific to Mexico; external findings (Paul et al., Priyadarshi et al.) described as suggestive but not directly extrapolable. |
| **OTHER INFORMATION** | | | |
| 22 | Funding | Give the source of funding and the role of the funders for the present study and, if applicable, for the original study on which the present article is based. | Funding section: “The authors received no financial support for the research, authorship, and/or publication of this article.” |

**Note:** This checklist was completed based on the revised manuscript (April 24, 2026). The study design is ecological (cross-sectional at state level); STROBE items applicable to individual-level cohort or case-control designs are not applicable here and are noted accordingly.
